# Butyrate as a Potential Modulator in Gynecological Disease Progression

**DOI:** 10.3390/nu16234196

**Published:** 2024-12-04

**Authors:** Nayeon Kim, Changwon Yang

**Affiliations:** Department of Science Education, Ewha Womans University, Seoul 03760, Republic of Korea; nayeon7112@ewha.ac.kr

**Keywords:** gynecological diseases, short-chain fatty acids, butyrate, microbiota, high-fiber diet, cervical cancer, ovarian cancer, endometrial cancer, polycystic ovary syndrome, endometriosis

## Abstract

This review investigates the therapeutic potential of butyrate, a short-chain fatty acid (SCFA) produced by gut microbiota, in the prevention and treatment of various gynecological diseases, including polycystic ovary syndrome (PCOS), endometriosis, and gynecologic cancers like cervical and ovarian cancer. These conditions often pose treatment challenges, with conventional therapies offering limited and temporary relief, significant side effects, and a risk of recurrence. Emerging evidence highlights butyrate’s unique biological activities, particularly its role as a histone deacetylase (HDAC) inhibitor, which allows it to modulate gene expression, immune responses, and inflammation. In PCOS, butyrate aids in restoring hormonal balance, enhancing insulin sensitivity, and reducing chronic inflammation. For endometriosis, butyrate appears to suppress immune dysregulation and minimize lesion proliferation. Additionally, in cervical and ovarian cancers, butyrate demonstrates anticancer effects through mechanisms such as cell cycle arrest, apoptosis induction, and suppression of tumor progression. Dietary interventions, particularly high-fiber and Mediterranean diets, that increase butyrate production are proposed as complementary approaches, supporting natural microbiota modulation to enhance therapeutic outcomes. However, butyrate’s short half-life limits its clinical application, spurring interest in butyrate analogs and probiotics to maintain stable levels and extend its benefits. This review consolidates current findings on butyrate’s multifaceted impact across gynecological health, highlighting the potential for microbiota-centered therapies in advancing treatment strategies and improving women’s reproductive health.

## 1. Introduction

Gynecological diseases, referring to disorders of the female reproductive system, pose significant public health and societal challenges. This broad category includes a variety of benign and malignant tumors as well as endocrine disorders, including polycystic ovary syndrome (PCOS) and endometriosis. These conditions profoundly affect women’s quality of life, impacting their physical health, emotional well-being, and reproductive capabilities. Unfortunately, most current therapies offer only temporary relief, often come with undesirable side effects, may interfere with the ability to conceive, and carry a risk of symptom recurrence after discontinuation. In the management of various health conditions, especially gynecological disorders, proactive measures can significantly reduce the incidence and severity of diseases.

Nutrition plays a critical role in managing gynecological diseases through its impact on hormonal balance, inflammation, and metabolic health [1]. Diets rich in vitamins, minerals, and antioxidants can help modulate disease symptoms by reducing oxidative stress and inflammation. Specific dietary patterns, like the Mediterranean diet, and nutrients, such as vitamin D, omega-3 fatty acids, and flavonoids, have shown promise in alleviating symptoms and potentially lowering disease risk. A high intake of red meat, alcohol, and trans fats, on the other hand, has been linked to an increased risk of these disorders. This underscores the potential of tailored dietary interventions as a complementary approach to conventional treatments. Numerous epidemiological and experimental studies have shown that diet, particularly the intake of dietary fiber, plays a crucial role in cancer prevention. Dietary fiber not only aids in digestion but also promotes a healthy gut microbiome, which can reduce inflammation and lower the risk of certain cancers like colorectal cancer. High-fiber diets help in binding potential carcinogens in the digestive tract and facilitate their excretion, thereby decreasing exposure to harmful substances. Therefore, incorporating adequate dietary fiber is considered an important strategy in reducing cancer risk [2].

Recent advancements have led to comprehensive efforts aimed at fully characterizing the microbiota across various human body sites under different health and disease conditions. High-throughput sequencing technologies have enabled researchers to delve deeper into the complex communities of bacterial microorganisms inhabiting organs such as the gut, skin, oral cavity, and reproductive tract. By shedding light on the symbiotic relationship between humans and their resident microbiota, these studies underscore the importance of bacterial microorganisms in influencing host metabolism and maintaining homeostasis [3]. Novel therapeutic approaches, such as using prebiotics, probiotics, and dietary interventions to restore microbial balance, could serve as promising strategies for preventing and treating gynecological diseases. The microbiomes of the gut and female reproductive tract form intricate biological ecosystems that engage in ongoing bidirectional communication. Especially, the gut and cervicovaginal microbiotas are increasingly recognized for their ability to interact with other organs and significantly influence the host’s health and disease equilibrium [4]. These microbial communities can alter the homeostasis of distant organs and bodily systems beyond their primary locations. This interaction between the two microbial communities plays a crucial role in maintaining the balance of various physiological processes, with significant effects on women’s overall health [5].

**Figure 1:**
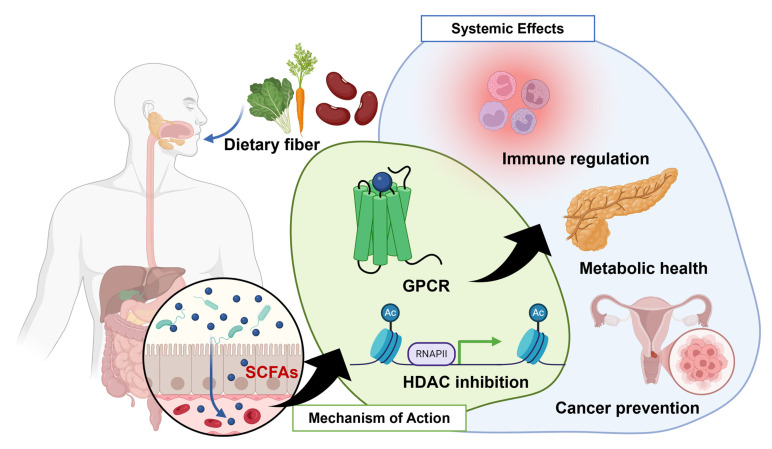
The systemic effects of dietary fiber and SCFAs on health. Dietary fiber, primarily from vegetables, legumes, and whole grains, is fermented by gut microbiota to produce short-chain fatty acids (SCFAs), including acetate, propionate, and butyrate. These SCFAs interact with G-protein-coupled receptors (GPCRs) on host cells, initiating signaling pathways which contribute to various physiological benefits. SCFAs also inhibit histone deacetylase (HDAC) activity, leading to changes in gene expression which support immune regulation, reduce inflammation, and maintain metabolic health. Additionally, the systemic effects of SCFAs play a role in cancer prevention by modulating cellular proliferation and apoptosis, particularly in reproductive systems.

As food passes through the colon, undigested dietary fibers serve as substrates for fermentation by the gut microbiota. This microbial fermentation process produces short-chain fatty acids (SCFAs) such as acetate, propionate, and butyrate. These SCFAs play multifaceted roles in both the gut microbiota and intestinal epithelial cells [6]. SCFAs affect cellular functions primarily via two key mechanisms (Figure 1). First, they serve as ligands for specific G-protein-coupled receptors [7]. Acetate primarily activates GPR43, while butyrate is a potent activator of GPR41 [8]. Propionate has the unique ability to effectively activate both GPR41 and GPR43. These receptor interactions are crucial for mediating the physiological effects of SCFAs, including their roles in metabolism, immune regulation, and the gut–brain axis [9]. Second, SCFAs act as inhibitors of histone deacetylase (HDAC), an enzyme involved in chromatin remodeling and the epigenetic regulation of gene expression. Inhibition of HDAC by SCFAs leads to increased histone acetylation, resulting in altered gene transcription which can influence processes such as cell proliferation, differentiation, and apoptosis [10]. Anaerobic bacteria, particularly in mucosal environments such as the gastrointestinal tract and the female genital tract during bacterial vaginosis, are prolific producers of SCFAs. Studies have confirmed the presence of multiple SCFAs in the lower genital mucosa [11]. These findings suggest that SCFAs contribute to the immunological milieu of the female reproductive tract. Moreover, SCFAs play an essential role in regulating metabolism during pregnancy [12]. The composition of the maternal microbiota and the production of SCFAs shift significantly during pregnancy, affecting both the mother and the developing fetus’s metabolic health. While substantial evidence indicates that SCFAs are involved in various aspects of female reproduction, a comprehensive understanding of their specific roles in gynecological diseases is still lacking.

In the context of gynecological diseases, the effects of SCFAs like acetate and propionate have been documented, but most research focuses on the benefits of butyrate. Butyrate is a naturally occurring SCFA with four carbon atoms, produced in the colon through the bacterial fermentation of dietary fiber consumed in the diet. An estimated 225 bacterial species are capable of producing butyrate. There is considerable variation in butyrate production capacities among different bacterial families. The most significant contributors are specific families within the phylum Firmicutes, though other phyla—including Actinobacteria, Bacteroidetes, Fusobacteria, Proteobacteria, Spirochaetes, and Thermotogae—also play important roles [13]. Butyrate plays a significant role in maintaining colon health and serves as an energy source for colonocytes [13]. Butyrate supports colon health by maintaining epithelial integrity, promoting cellular homeostasis, and exerting anti-inflammatory, antioxidant, and anticancer effects [14]. Among the numerous hypotheses regarding butyrate’s mechanisms of action, one theory stands out due to its significant impact: butyrate’s ability to function as an HDAC inhibitor [15]. This capacity enables butyrate to modulate gene expression by promoting histone acetylation, which leads to a more relaxed chromatin structure and enhances transcriptional accessibility. Owing to their relatively low toxicity, HDAC inhibitors are considered promising candidates for cancer therapy, especially when used in combination with other chemotherapeutic agents. Their ability to modulate gene expression epigenetically allows them to target cancer cells effectively, potentially enhancing the efficacy of existing treatments and overcoming drug resistance [16]. The therapeutic effects of butyrate have been predominantly documented in diseases located near its primary site of production, such as colorectal cancer. Patients with colorectal cancer often exhibit lower levels of butyrate-producing microbes compared to healthy individuals. Additionally, an inverse correlation has been observed between tumor size and fecal butyrate concentrations in these patients, implying that higher levels of butyrate may inhibit tumor growth and progression [17]. Emerging research has begun to highlight the presence and potential role of butyrate within the female reproductive tract. Despite these advancements, there is a notable lack of comprehensive reviews focusing on butyrate’s role in the amelioration of gynecological diseases. This review aims to fill that gap by discussing evidence that suggests that enhancing butyrate production through dietary interventions could serve as a promising strategy for the prevention and treatment of gynecological conditions. By exploring butyrate’s mechanisms of action beyond the gut, we hope to shed light on novel therapeutic approaches that could improve women’s reproductive health.

## 2. Role of Gut Microbiota in Female Reproductive Health and Disease

The gut microbiota plays a critical role in regulating immune responses and metabolic processes, which are essential for maintaining the body’s homeostasis. Disruptions in the balance of these microbial communities—a condition known as dysbiosis—have been linked to a wide range of health disorders both within the gastrointestinal tract and in other organ systems [18]. Alterations in the gut microbiota can significantly impact the female reproductive system and the health of offsprings [19,20]. The gut microbiome plays a crucial role in regulating hormonal balance, immune function, and metabolic processes, all of which are essential for reproductive health. Moreover, during pregnancy, the maternal gut microbiota influences fetal development by modulating nutrient availability [21]. Changes in the maternal microbiome can affect the transfer of essential metabolites and microbial components to the fetus, thereby impacting the offspring’s immune programming and susceptibility to diseases later in life. The gut microbiota also plays a crucial role in promoting the maturation and development of the immune system during infancy. Specifically, it facilitates the maturation of intestinal immune cells such as CD4^+^ helper T cells, CD8^+^ cytotoxic T cells, and dendritic cells [22]. Moreover, studies have shown that maternal gut dysbiosis is associated with adverse pregnancy outcomes such as preeclampsia, gestational diabetes, and preterm birth [23,24,25].

Although the gut microbiome represents a distinct microbial ecosystem separate from that of the female reproductive tract, it exhibits clear sexual dimorphism in its composition and functional dynamics [26]. This inherent sex-based variation underscores the importance of integrating sex-specific microbiome analyses into research on human health and disease. Historically, the uterine cavity—the upper reproductive tract—was considered a sterile environment, free from microbial inhabitants. In contrast, the lower reproductive tract, specifically the cervicovaginal region, is known to host a vast and diverse microbiota comprising trillions of bacteria [4]. In a study involving 132 pregnant women, researchers found that 36% of the participants had identical bacterial species present in both their rectum and vagina [27]. Moreover, 68% of these bacterial species shared identical genotypes between the two sites. This significant overlap in microbial composition indicates not only the presence of similar bacterial species but also comparable cell densities of each species in both anatomical niches. Both the gut and vaginal microbiomes have been shown to harbor five common bacterial phyla: Firmicutes, Bacteroidetes, Proteobacteria, Actinobacteria, and Fusobacteria. These findings highlight a strong correlation between gut and vaginal microbiotas, suggesting potential microbial transmission or shared environmental factors influencing both communities. This overlap suggests a potential microbial exchange between these two niches, highlighting the interconnectedness of the body’s microbial ecosystems [28]. Emerging research indicates that the gut microbiota may indirectly influence the development of cervical cancer by affecting the vaginal microbiota. One possible mechanism is the migration of microbes from the gut to the vagina, suggesting that the gut serves as a reservoir for many vaginal microorganisms. Substantial overlap not only demonstrates the co-existence of microorganisms in both anatomical sites but also highlights a strong correlation in bacterial density and diversity between these two ecological niches [29]. An imbalance in these microbial communities, particularly the overgrowth of pathogenic bacteria in the vagina, can lead to chronic cervical inflammation. This persistent inflammatory state creates a conducive environment for carcinogenic transformations in the cervical mucosa by promoting DNA damage, altering cellular proliferation, and impairing immune responses [30].

Emerging research indicates that manipulating both the vaginal and gut microbiomes can offer promising therapeutic strategies for addressing various gynecologic conditions, including bacterial vaginosis, PCOS, and endometriosis [31]. Approaches such as probiotics, prebiotics, dietary interventions, and microbiota transplants are being explored to restore microbial balance and reduce inflammation. These interventions have shown potential in improving reproductive health by influencing hormone regulation, immune responses, and the microbial ecosystem in both the gut and reproductive tract. Despite the close anatomical proximity between the cervicovaginal space and the uterine cavity, the healthy uterine cavity has traditionally been considered a sterile environment. This belief stems from the assumption that the cervical mucus plug acts as an impermeable barrier, preventing bacteria from ascending from the vagina into the uterus [32]. However, recent research has challenged this notion, suggesting that the cervical mucus plug may not be completely impermeable to bacterial passage [33]. Consequently, the uterus may not be as sterile as previously thought. Investigating the microbiota of the upper female genital tract, particularly within the intrauterine and endometrial environments, presents significant challenges [34]. Research has indicated that certain bacterial species are present in both the upper and lower female genital tracts, suggesting some overlap in microbial communities [35]. However, it remains uncertain whether identical bacterial species interact differently with the endometrial mucosa compared to their interactions within the vagina or cervix. Emerging studies increasingly demonstrate that the immune responses within the endometrium can be influenced by the microbiota residing in the upper female genital tract and the uterine environment [36]. This growing body of evidence underscores the potential role of the uterine microbiome in modulating host immune functions and maintaining reproductive health. These findings reveal the importance of employing stringent and validated methodologies for transvaginal sampling to accurately characterize the microbiome of the upper female reproductive tract, thereby minimizing contamination from the vaginal microbiota [37]. The microbiome also undergoes significant changes, termed oncobiosis, in ovarian cancer, with altered bacterial populations across various bodily compartments, such as the peritoneum, upper and lower genital tract, and intestines [38].

Disruptions in the production of steroid hormones circulating within the host can initiate disease by causing the dysregulation of the microbiome [39,40]. Prolonged alterations in reproductive hormone levels can lead to significant shifts in the gut microbiota composition and function [41]. These hormonal imbalances can alter the gut environment, impacting microbial metabolism, immune responses, and intestinal barrier integrity. Recent evidence indicates that PCOS is closely associated with significant alterations in gut microbial diversity and composition. These changes in the intestinal microbiota are thought to contribute to the metabolic and hormonal imbalances characteristic of PCOS [42]. Moreover, manipulating the gut microbiota has been shown to influence PCOS phenotypes, suggesting that the microbiome plays a pivotal role in the syndrome’s pathogenesis and could be a target for therapeutic intervention [43]. Recognizing that alterations in the composition and function of the gut microbiota can profoundly impact the host’s metabolism and sex hormone production, researchers have undertaken studies to profile and compare the intestinal microbial communities of women with PCOS and healthy controls [44]. Women with PCOS exhibit distinct gut microbiota profiles, including reduced microbial diversity and shifts in specific bacterial taxa, which may contribute to the metabolic and hormonal imbalances characteristic of the syndrome [45]. Furthermore, in obese patients with PCOS, the gut microbiota differs significantly from that of patients with PCOS who are not obese and healthy controls [46]. Obesity in patients with PCOS is associated with reduced butyrate-producing bacteria, which may contribute to metabolic disturbances and exacerbate inflammation, impacting both gut health and systemic hormone regulation. Despite the increasing interest in the gut microbiota’s role in PCOS, relatively few studies have explored the impact of probiotics on gut microbiota composition and its metabolites in this condition. Notably, metagenomic techniques have been used to investigate how a specific probiotic strain could regulate sex hormone levels in women with PCOS [47]. SCFAs have been implicated in regulating metabolic processes and hormonal signaling pathways in PCOS conditions. This indicates that the gut microbiota and its metabolites might play a significant role in the pathophysiology of PCOS, potentially offering new avenues for therapeutic interventions.

Multiple studies have identified a significant association between microbiota composition and the pathogenesis of endometriosis. For instance, both mouse models and women diagnosed with endometriosis have been found to exhibit altered gut microbial communities [48]. Additionally, women with endometriosis are more likely than those without the condition to experience uterine microbial dysbiosis [49,50]. These alterations suggest that imbalances in the gut and uterine microbiota may play a crucial role in the development and progression of endometriosis, potentially through mechanisms involving immune modulation and chronic inflammation. Understanding these microbial influences could open new avenues for therapeutic interventions targeting the microbiota to manage or prevent endometriosis. Meanwhile, emerging evidence suggests that gut dysbiosis, characterized by reduced microbial diversity and imbalances in bacterial populations, may contribute to the pathogenesis of uterine fibroids (UFs) [51]. The altered microbiome influences estrogen metabolism, leading to a hyperestrogenic state, which is a key factor in fibroid development. Given that a variety of gynecological diseases are linked to alterations in the microbiota, modulating these microbial communities offers a promising strategy for disease mitigation. However, there is currently a lack of comprehensive understanding regarding how metabolites produced by microorganisms influence gynecological disease models. Butyrate, one of the most well-known SCFAs due to its beneficial biological activities, has been extensively studied in the context of gastrointestinal health but less so in gynecological diseases.

## 3. Metabolic Pathways of Butyrate Production

One mechanism by which mammalian gut bacteria exert a profound influence on host physiology and immunological processes is through the fermentation of otherwise indigestible dietary nutrients into biologically active metabolites. SCFAs, particularly butyrate, acetate, and propionate, are produced by gut microbiota through the fermentation of dietary fibers, promoting colon health and potentially reducing cancer risk [52]. The gut microbiota consists of a variety of bacterial species that contribute differently to the production of SCFAs in the intestinal lumen [53]. Specifically, Gram-negative bacteria such as those from the Bacteroides genus predominantly generate acetate and propionate. In contrast, Gram-positive bacteria belonging to the Firmicutes phylum mainly produce butyrate. Among these, *Faecalibacterium prausnitzii* is one of the most abundant commensal bacteria in the human gut and is recognized as a keystone species for its ability to produce butyrate via the acetyl-CoA pathway. This species thrives on dietary fiber and plays a significant role in shaping the gut microbiota composition [54]. Similarly, members of the genus *Roseburia*, such as *Roseburia intestinalis* and *Roseburia hominis*, are known for their strict anaerobic requirements and their capacity to ferment a wide range of carbohydrates, including fructose, glucose, and maltose, to generate butyrate [55]. *Eubacterium rectale* and *Eubacterium hallii* also contribute significantly to butyrate synthesis in the gut, particularly through cross-feeding interactions [56]. For instance, *Eubacterium hallii* utilizes lactate and acetate, metabolites produced by other bacteria like *Bifidobacterium*, to produce butyrate, thereby enhancing metabolic interconnectivity within the gut microbiota. Butyrate-producing bacteria, also known as butyrogenic bacteria, are essential members of the human gut microbiome that convert dietary carbohydrates into butyrate through metabolic pathways involving intermediates such as pyruvate, acetyl-CoA, and crotonyl-CoA. Their ability to perform this conversion in monoculture highlights their potential for therapeutic applications, such as probiotics aimed at restoring or enhancing butyrate production in the gut [57]. Although most butyrate-producing bacteria belong to the Firmicutes phylum, species from other phyla, such as Bacteroidetes and Actinobacteria, also contribute to butyrate production via alternative pathways like the glutarate and lysine pathways [13]. A higher abundance of these butyrate-producing bacteria is often associated with a healthy gut microbiome, while reduced levels have been linked to various gastrointestinal disorders, such as inflammatory bowel disease and colorectal cancer [58].

Butyrate biosynthesis in bacteria proceeds through four distinct metabolic pathways: the acetyl-CoA pathway, the glutarate pathway, the 4-aminobutyrate pathway, and the lysine pathway [13,59]. All these pathways converge at a critical energy-generating step involving the conversion of crotonyl-CoA to butyryl-CoA [60]. The acetyl-CoA pathway is predominantly utilized by Firmicutes, a major bacterial group responsible for butyrate production in the gut microbiota. In this pathway, acetyl-CoA is first condensed into acetoacetyl-CoA by thiolase. This intermediate is then reduced to 3-hydroxybutyryl-CoA by β-hydroxybutyryl-CoA dehydrogenase. The subsequent dehydration by crotonase yields crotonyl-CoA, which is finally reduced to butyryl-CoA by the butyryl-CoA dehydrogenase electron-transferring flavoprotein complex. In the glutarate pathway, glutarate undergoes oxidation to form 2-oxoglutarate, which is then reduced to 2-hydroxyglutarate by 2-hydroxyglutarate dehydrogenase. This compound is converted to 2-hydroxyglutaryl-CoA via glutaconate CoA transferase. The next step involves the oxidation to glutaryl-CoA by 2-hydroxyglutaryl-CoA dehydrogenase, followed by decarboxylation to crotonyl-CoA mediated by glutaconyl-CoA decarboxylase. The 4-aminobutyrate pathway begins with the conversion of 4-aminobutyrate into succinate semialdehyde. This intermediate is reduced to 4-hydroxybutyrate by 4-hydroxybutyrate dehydrogenase. Subsequently, 4-hydroxybutyrate is transformed into 4-hydroxybutyryl-CoA by butyryl-CoA:4-hydroxybutyrate CoA transferase. Dehydration of this molecule by 4-hydroxybutyryl-CoA dehydratase produces vinyl-acetyl-CoA, which is finally converted into crotonyl-CoA. The lysine pathway represents the fourth route for butyrate production. Lysine is initially converted into β-lysine by lysine 2,3-aminomutase. β-lysine is then rearranged into 3,5-diaminohexanoate by β-lysine 5,6-aminomutase. The deamination of this compound by 3,5-diaminohexanoate dehydrogenase yields the keto acid 3-keto-5-aminohexanoate. This intermediate reacts with acetyl-CoA in a reaction catalyzed by the 3-keto-5-aminohexanoate cleavage enzyme, producing 3-aminobutyryl-CoA and acetoacetate. Finally, 3-aminobutyryl-CoA is converted into crotonyl-CoA by 3-aminobutyryl-CoA ammonia lyase.

The conversion of butyryl-CoA to butyrate involves two primary metabolic routes utilized by gut bacteria. The first route involves the phosphorylation of butyryl-CoA to butyryl-phosphate, catalyzed by phosphotransbutyrylase [61]. Butyryl-phosphate is then dephosphorylated into butyrate by butyrate kinase. This pathway is significant in bacterial species that produce butyrate as a major fermentation end product. The second route employs a CoA-transferase mechanism, where butyryl-CoA reacts with acetate in a reaction facilitated by butyryl-CoA-transferase, resulting in the formation of butyrate and regenerating acetyl-CoA. Understanding these metabolic pathways is crucial as they highlight the diverse mechanisms by which the gut microbiota contributes to butyrate production. Elucidating these pathways enhances our comprehension of how microbial metabolism influences cell physiology, which will be further explored in the subsequent section.

## 4. Role of Butyrate in Gynecological Diseases

Recent research has detected various microbial metabolites in animal umbilical cord blood, placenta, fetal intestine, fetal brain, and even in the human fetal intestine [62,63,64]. This emerging evidence suggests that microbial metabolites produced by the maternal gut microbiota can cross the placental barrier and interact with fetal tissues. These discoveries shed light on the potential mechanisms by which maternal microbiota can affect fetal development, possibly mediating immune system maturation, metabolic programming, and organogenesis through their metabolites. Maternal-associated microbial metabolites, such as SCFAs, tryptophan derivatives, and bile acid derivatives, can play a pivotal role in the development of offspring diseases. These metabolites are transferred to the fetus during pregnancy or via breastfeeding, influencing immune system development and disease susceptibility. Studies have linked maternal microbiota and its metabolites to conditions like asthma, type 1 diabetes, food allergies, and autism spectrum disorder in offsprings [65]. In particular, SCFAs can stimulate oxidative bursts in neutrophils, leading to the rapid release of reactive oxygen species which are crucial for microbial killing [11]. Consequently, SCFAs, either independently or synergistically with other microbial products, have the ability to recruit and activate innate immune cells within the female reproductive tract. In the reproductive system, an imbalance of SCFAs is linked to conditions like bacterial vaginosis and other gynecological issues by disrupting the vaginal pH and contributing to inflammation [66]. For instance, serum levels of butyrate, acetate, and propionate were significantly reduced in women who developed late-onset preeclampsia compared to pregnant control subjects [67]. Furthermore, studies have demonstrated that fecal concentrations of SCFAs were elevated in the control group of pregnant women, whereas the levels of butyrate were markedly decreased in those with preeclampsia [68]. Research using rat models of preeclampsia discovered that the oral administration of SCFAs, specifically propionate and butyrate, led to a significant decrease in blood pressure [69]. Additionally, these treatments enhanced placental function and promoted better embryonic development. Moreover, the administration of butyrate during pregnancy and nursing has been shown to attenuate the progression of type 1 diabetes mellitus in female offspring of non-obese diabetic mice [70]. Despite these observed physiological effects of butyrate in the female reproductive system, our understanding of its role in gynecological diseases remains limited and not well organized. The mechanisms by which butyrate influences gynecological health are not fully elucidated, and existing studies often provide fragmented or preliminary insights. Comprehensive research is needed to systematically investigate the effects of butyrate on gynecological conditions such as endometriosis, PCOS, and gynecological cancers. A deeper understanding of these mechanisms could potentially lead to novel therapeutic strategies that harness butyrate’s beneficial properties for the prevention and treatment of gynecological diseases.

SCFAs produced within the gut lumen are predominantly absorbed by the intestinal mucosa [71]. Within the cecum and large intestine, approximately 95% of SCFAs are swiftly absorbed by colonic epithelial cells, while the remaining 5% are eliminated through fecal excretion [72]. Directly measuring butyrate levels in humans is challenging due to its rapid absorption and metabolism in the colon. This efficient uptake allows butyrate to exert significant physiological effects, influencing not only local gut health but also systemic immune responses. Several lines of evidence suggest that butyrate exerts its effect through its ability to inhibit HDAC. The regulation of histone acetylation is a fundamental epigenetic mechanism that plays a pivotal role in controlling gene expression patterns, thereby influencing cellular differentiation and the biological phenotype [73]. The epigenetic regulation of gene expression through HDAC inhibition makes this mechanism particularly compelling in understanding and combating diseases where these processes are disrupted [74]. In recent years, HDAC inhibitors have gained significant attention for their ability to promote cell differentiation, halt cell growth, and trigger apoptosis [75]. By modulating chromatin structure and gene transcription, HDAC inhibitors can influence cell fate and are being explored as promising therapeutic agents in cancer treatment. Among these HDAC inhibitors, a naturally occurring compound, sodium butyrate (NaB)—the sodium salt of butyric acid—has been extensively studied due to its wide range of beneficial properties. NaB not only enhances intestinal barrier integrity and reduces inflammation but also promotes apoptosis in cancer cells by modulating gene expression [76]. NaB has been shown to inhibit tumor growth, improve the efficacy of chemotherapy and radiotherapy, and restore the balance of gut microbiota. The NaB-induced hyperacetylation of histones leads to the relaxation of chromatin structure, facilitating the transcriptional activation of the gene encoding p21. The p21 protein serves as a cyclin-dependent kinase inhibitor, specifically targeting cyclin E-CDK complexes. This inhibition effectively halts the progression of cells from the G1 to the S phase of the cell cycle, resulting in cell cycle arrest at the G1 phase. Importantly, this mechanism operates independently of the tumor suppressor protein p53, suggesting an alternative pathway for regulating cell proliferation [15]. Given these multifaceted biological activities of butyrate and its derivative NaB, particularly their roles in cell cycle regulation, apoptosis, and modulation of gene expression through epigenetic mechanisms, it becomes imperative to explore their potential effects in gynecological diseases. Meanwhile, many cancers are either inherently resistant to chemotherapy or develop resistance after an initial partial response, enabling them to evade the cytotoxic effects of treatment [77]. This ability to resist chemotherapy is a major hurdle in the successful management of cancer. Chemoresistance not only diminishes the effectiveness of standard treatments but also contributes to cancer recurrence and progression. Overcoming this obstacle requires a deeper understanding of the underlying mechanisms of resistance, such as drug efflux, DNA repair enhancement, and apoptosis inhibition. Clinical trials are exploring the use of HDAC inhibitors as genetic regulators for chemoprevention, particularly in patients who have a high risk of relapse after specific therapies or when combined with other treatment regimens for certain advanced-stage cancers [78]. By modulating gene expression, HDAC inhibitors like butyrate can enhance the effectiveness of conventional treatments and potentially reduce the likelihood of cancer recurrence. The oral administration of butyrate or its analogs presents a promising strategy for alternative treatments or sensitizing tumor cells in conjunction with drugs commonly used in chemotherapy. Therefore, understanding how NaB influence cellular processes in the female reproductive system could unveil new therapeutic avenues for conditions such as endometriosis, PCOS, and gynecological cancers. The subsequent sections will delve into the known effects of NaB in various gynecological disorders, highlighting current research findings and potential applications.

### 4.1. Cervical Cancer

Cervical cancer is the fourth most frequently diagnosed cancer among women worldwide, representing a significant global health issue [79]. Despite its high incidence, the 5-year survival rate for patients with cervical cancer remains disappointingly low. Although different in developed and developing countries, cervical cancer has the highest incidence and mortality rate among gynecological cancers [80]. High-risk human papillomavirus (HPV) contributes to cervical cancer by expressing viral oncogenes E6 and E7, which are necessary and sufficient for cells to develop and maintain malignant characteristics. After HPV DNA integrates into the host genome, these viral oncoproteins are continuously produced and can interact with enzymes that modify histones, such as histone acetyltransferases (HATs) and HDACs [81]. By altering the acetylation status of histones, HDACs cause chromatin condensation, which silences gene expression and facilitates oncogenic processes [82]. A key feature of HPV-induced transformation is the post-translational interaction of E6 and E7 proteins with cellular regulators of the cell cycle: E6 interacts with p53 and triggers its degradation through the ubiquitin–proteasome system. Cisplatin-based chemotherapy is a standard treatment widely used for patients with cervical cancer. However, despite its common application, many patients still face a poor prognosis due to factors such as tumor recurrence, severe side effects, and the development of resistance to chemotherapeutic drugs. These challenges underscore the need for novel therapeutic strategies and combination treatments to enhance efficacy, overcome drug resistance, and improve overall patient survival rates in cervical cancer management [83].

Regarding the role of SCFAs in cervical cancer, studies have shown that propionate induces apoptosis in HeLa cervical cancer cells [84]. Propionate’s anticancer effect is primarily mediated through the production of reactive oxygen species, mitochondrial dysfunction, and inhibition of the AKT/mTOR and NF-κB pathways. However, in cervical cancer, the effects of SCFAs have been more extensively studied and are better understood for butyrate. Dyson et al. found that concentrations of NaB above 0.5 mM cause the cell death of cervical cancer cells, while lower concentrations reduce cell proliferation without causing death. The effects on cell proliferation are dose-dependent, with butyrate concentrations also leading to an increased cell size and cytokeratin synthesis, making NaB a potential agent for combined cancer treatments [85]. Moreover, NaB induces cell cycle arrest during the transition from the G1 to S phase in HPV-positive cervical cancer cells by upregulating CDK inhibitors like p21 and p27, while suppressing CDK2 activity [86]. Additionally, it promotes apoptosis even in the presence of active HPV oncogenes, making it a promising agent for targeting HPV-driven tumors.

The reduction in pro-survival proteins BCL-2 and BCL-X_L_ contributes to the commitment of cells to undergo apoptosis. While these anti-apoptotic members of the BCL-2 family regulate the mitochondrial death pathway, the tumor suppressor p53 likely plays a pro-apoptotic role in the mitochondria. p53 can directly activate pro-apoptotic proteins like BAX or bind to BCL-2 and BCL-X_L_, inhibiting their function. This leads to the release of cytochrome c and the activation of caspases, ultimately triggering programmed cell death [87]. NaB enhances apoptosis in HeLa cells by triggering the release of cytochrome c and the apoptosis-inducing factor from the mitochondria. This effect is further amplified when telomerase is inhibited, suggesting that NaB, in combination with telomerase inhibition, could be a potent strategy for inducing apoptosis in cancer cells via the mitochondrial pathway [88]. Another study also found that NaB inhibits the proliferation, migration, and invasion of cervical cancer cells by inducing mitochondria-dependent apoptosis. It achieves this by inhibiting mitochondrial complex I, leading to a reduction in NADH and NAD^+^ levels, which triggers oxidative stress and promotes the release of cytochrome c and the activation of caspase-9. These findings suggest that butyrate’s ability to disrupt mitochondrial function plays a crucial role in its anticancer effects [89].

**Figure 2:**
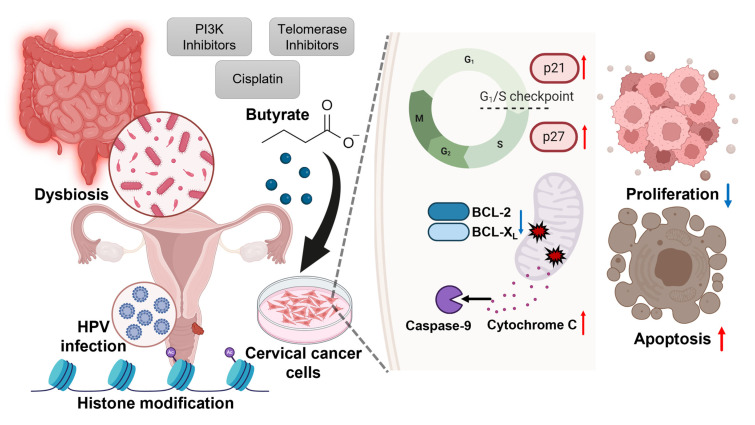
The role of butyrate in modulating cervical cancer cell proliferation and apoptosis. Dysbiosis in the gut microbiota leads to altered microbial composition, which can impact cervical health and increase susceptibility to infections such as HPV. HPV infection promotes histone modification, affecting gene expression and contributing to cancer cell growth. Butyrate exerts anticancer effects on cervical cancer cells by regulating the cell cycle and inducing apoptosis. In the presence of butyrate, cell cycle regulators p21 and p27 are upregulated, leading to G1/S phase arrest, which inhibits cell proliferation. Butyrate also enhances mitochondrial apoptosis by downregulating anti-apoptotic proteins. Butyrate also leads to the release of cytochrome c and the activation of caspase-9, which, in turn, promotes cell death. The therapeutic potential of butyrate is enhanced when combined with treatments like PI3K inhibitors, telomerase inhibitors, or cisplatin. Upward-pointing arrows represent the upregulation of the indicated proteins or cellular processes, while downward-pointing arrows indicate their downregulation.

Recently, it has been suggested that strategies to increase butyrate-producing strains may be useful in inhibiting cervical cancer metastasis [90]. Treatment with butyrate inhibits migration and invasiveness in HeLa cells. Elevated PI3K activity plays a significant role in promoting the proliferation and survival of various cancers, including cervical cancer. When combined with PI3K inhibitors, NaB’s ability to promote cell death is enhanced, particularly through the activation of caspase-3 and caspase-9 and the upregulation of CDK inhibitors. These findings suggest that targeting the PI3K pathway in conjunction with NaB could offer a promising strategy for treating cervical cancer [91]. Furthermore, the combination of NaB with 7-hydroxy-staurosporine (UCN-01) significantly amplifies its pro-apoptotic effects, promoting cell cycle arrest and apoptosis through the modulation of BCL-2 family proteins and the activation of p53 and p73 pathways [92]. Furthermore, NaB enhances cisplatin-induced apoptosis in cervical cancer cells by promoting mitochondrial dysfunction and increasing caspase activation. Moreover, while NaB alone can induce migration and partial epithelial–mesenchymal transition (EMT), its combination with cisplatin reverses these effects, reducing cell migration and invasion, thus enhancing the therapeutic potential of cisplatin [93]. In conclusion, butyrate exhibits potent anticancer effects in cervical cancer by inducing mitochondrial dysfunction, regulating the cell cycle, and promoting apoptosis, especially when used in combination with other therapeutic agents (Figure 2). These findings suggest the potential development of novel butyrate-based treatment strategies for cervical cancer and highlight the need for further research for clinical application.

### 4.2. Ovarian Cancer

Due to the absence of specific early symptoms, many patients with ovarian cancer are diagnosed at an advanced stage of the disease. This late detection leads to a high mortality rate, largely attributed to the recurrence of drug-resistant tumors and the poor response to salvage chemotherapeutic regimens. The persistence of chemoresistant disease underscores the urgent need for improved diagnostic methods and the development of more effective therapeutic strategies. Resistance to platinum-based chemotherapy is a significant hurdle in the treatment of ovarian cancer, often arising from multiple cellular mechanisms. Patients who experience a relapse within six months after completing first-line platinum therapy are classified as platinum-resistant [94]. This resistance develops in approximately 30–40% of women with ovarian cancer, leading to a poor prognosis and limited treatment options. The emergence of resistance to chemotherapeutic agents diminishes treatment efficacy, leading to disease recurrence and progression, and ultimately contributes to increased mortality among patients with ovarian cancer. Emerging evidence suggests that altered interactions between the host and its microbiota may play a significant role in the development of ovarian cancer. Importantly, species of *Lactobacilli* are recognized as protective agents against ovarian cancer [95]. Research indicates that vaginal microbial communities lacking *Lactobacillus* are more commonly found in patients with ovarian cancer compared to healthy controls. The alteration of the vaginal microbiota, especially the decrease in beneficial *Lactobacillus* species, may disrupt the local immune environment and contribute to carcinogenesis in ovarian tissues. A study utilizing TLR5-deficient mice, which exhibit features of a dysbiotic gut environment, has provided insights into this potential [96]. The absence of TLR5 leads to an imbalance in the gut microbiota, resulting in chronic inflammation mediated by tumor-promoting immune responses. This inflammation can facilitate malignant progression in distant organs, including the ovaries. These findings highlight the importance of the gut–immune axis and suggest that dysbiosis-induced inflammation may contribute to ovarian cancer pathogenesis.

Bacterial metabolic pathways involved in the production of SCFAs are upregulated in ovarian cancer tumors [97,98]. The enhanced activity of these pathways suggests a potential role of gut microbiota-derived metabolites in tumor progression, highlighting the importance of microbial interactions in ovarian carcinogenesis. Moreover, HDACs are markedly overexpressed in ovarian carcinoma tissues, suggesting a potential role for butyrate as an HDAC inhibitor [99]. This upregulation plays a pivotal role not only in the initiation and progression of carcinogenesis but also in the development of resistance to chemotherapeutic agents. NaB induces growth arrest and senescence-like phenotypes in ovarian cancer cells by causing a G0/G1 phase block [100,101]. The mechanism involves the upregulation of p21, leading to the dephosphorylation of Rb, which inhibits cell cycle progression, even in cells with mutated p53 genes. Moreover, butyrate has been shown to inhibit the proliferation of ovarian cancer cells by inducing apoptosis, even though it does not affect telomerase activity [102]. Furthermore, butyrate has been shown to significantly reduce the viability of ovarian cancer cells, such as OC-109 and SKOV3, through a dose- and time-dependent inhibition of cell growth [103]. NaB, in combination with calpeptin, was shown to significantly inhibit the growth and motility of ovarian cancer cells by inducing cell cycle arrest, apoptosis, and autophagy [104]. Furthermore, NaB exhibits significant anticancer effects by inducing the expression of epithelial markers like E-cadherin while inhibiting mesenchymal markers such as vimentin in ovarian cancer cells [105]. NaB also plays a crucial role in inducing ferroptosis in ovarian cancer cells by upregulating the expression of ARHGAP10, a protein which promotes this form of cell death [106]. ARHGAP10 overexpression, driven by NaB, decreases cell viability and increases the accumulation of lipid-based ROS and iron content, hallmarks of ferroptosis.

Approximately 20% of ovarian cancer relapses are refractory to platinum-based chemotherapy, resulting in a very poor prognosis for these patients. Acquired drug resistance has been extensively studied using various cisplatin-resistant ovarian cancer cell lines [107]. Multiple molecular mechanisms contributing to this resistance have been identified, including reduced intracellular accumulation of the drug due to impaired uptake or increased efflux, enhanced DNA repair mechanisms which counteract cisplatin-induced DNA damage, and alterations in DNA damage response pathways [108]. In vitro experiments using SKOV3 and OVCAR3 epithelial ovarian cancer cell lines have confirmed the correlation between HDAC overexpression and resistance to cisplatin [109]. These findings suggest that HDACs are critical mediators of chemoresistance in ovarian cancer, and targeting them could enhance the efficacy of existing therapeutic strategies.

Moreover, the combination of NaB and paclitaxel significantly increased cell death compared to either treatment alone, promoting apoptosis through the activation of the caspase cascade, independent of p53 status [110]. These findings suggest that NaB may serve as an effective adjuvant therapy in ovarian cancer, potentially allowing for lower doses of paclitaxel and reducing the associated side effects. In combination with aspirin, butyrate demonstrates a synergistic effect, significantly enhancing its anticancer activity, as indicated by experiments conducted on A2780 ovarian cancer cells [111]. Additionally, butyrate alters the expression of key drug resistance genes, increasing MDR1 expression while decreasing MRP1 and MRP2, which may contribute to its therapeutic potential in overcoming chemotherapy resistance in ovarian cancer [103]. In combination with haemanthamine, NaB induces a pronounced increase in histone H3 and H4 acetylation, which is more prominent in cancerous A2780 ovarian cells than in non-cancerous MRC-5 fibroblasts [112]. Furthermore, this combination causes a marked reduction in the viability and proliferation of A2780 cells, while the impact on non-cancer cells remains limited. The combination also results in the suppressed phosphorylation of checkpoint kinases Chk1 and Chk2, which may further enhance its selective cytotoxic effects against cancer cells.

Collectively, these studies highlight the significant anticancer potential of butyrate in ovarian cancer treatment. By modulating key pathways involved in cell cycle regulation, apoptosis, autophagy, epithelial–mesenchymal transition, and ferroptosis, butyrate exerts multifaceted effects that inhibit tumor growth and progression. The upregulation of bacterial metabolic pathways producing SCFAs and the overexpression of HDACs in ovarian cancer tissues underscore the importance of exploring gut microbiota-derived metabolites like butyrate as therapeutic agents. These findings suggest that butyrate and its derivatives could be promising candidates for developing novel treatments for ovarian cancer, warranting further preclinical and clinical studies to elucidate their efficacy and underlying mechanisms.

### 4.3. Endometrial Cancer

Endometrial cancer, originating from the inner lining of the uterus, is one of the most frequently diagnosed malignant tumors of the female reproductive system. It significantly threatens women’s health worldwide, contributing to substantial morbidity and mortality rates [113]. Elevated estrogen levels can lead to significant alterations in the vaginal microbiome, which may indirectly promote the development of endometrial carcinoma by disrupting the gut–vaginal microbiome axis [114]. Notably, a systematic review and meta-analysis found that a higher dietary fiber intake is associated with a reduced risk of endometrial cancer [115]. This association suggests that increasing the consumption of fiber-rich foods may have a protective effect against the development of endometrial carcinoma, potentially through mechanisms involving SCFA production and the modulation of hormonal and inflammatory pathways.

Saito and colleagues have investigated the dose-dependent effects of NaB on human endometrial adenocarcinoma cells, demonstrating its ability to induce cell cycle arrest, particularly in the G1 phase, by inhibiting DNA synthesis [116]. NaB significantly suppressed the growth of human endometrial cancer cells, independent of their p53 gene status [100]. NaB, along with other HDAC inhibitors, was shown to induce cell cycle arrest in the G1 phase and stimulate apoptosis by modulating the expression of key regulatory proteins such as p21, p27, and Bcl-2 [117]. These findings suggest that NaB’s ability to inhibit cancer cell growth through chromatin remodeling and gene expression regulation, positioning it as a promising therapeutic agent for targeting endometrial cancer. Furthermore, NaB significantly inhibits the self-renewal capacity of endometrial cancer stem-like cells by inducing DNA damage and promoting the production of ROS. Treatment with NaB resulted in the suppression of colony formation and a marked increase in DNA damage markers such as γH2AX, suggesting heightened sensitivity of these cancer stem-like cells to butyrate-induced damage [118]. NaB also induces ferroptosis in endometrial cancer cells by targeting the RBM3/SLC7A11 axis. NaB promotes the expression of RBM3, which in turn downregulates SLC7A11, a key component involved in glutathione synthesis, thereby triggering ferroptosis through oxidative stress and lipid peroxidation [119]. Furthermore, NaB enhances the cytotoxic effects of adriamycin in uterine cancer cells by promoting apoptosis through the downregulation of hTERT, a key component of telomerase [120]. The combination of NaB and adriamycin significantly reduced cell viability, induced caspase activation, and inhibited telomerase activity, which is critical for cancer cell immortality. Collectively, these studies highlight the multifaceted anticancer mechanisms of NaB in endometrial carcinoma cells, reinforcing its potential as a promising therapeutic agent which targets various pathways to inhibit tumor growth and progression.

### 4.4. PCOS

PCOS is a prevalent endocrine disorder affecting approximately 6% to 20% of women of reproductive age worldwide, with the exact prevalence varying based on the diagnostic criteria used [121]. PCOS is a complex and heterogeneous condition with a spectrum of clinical presentations. This complex syndrome is characterized by a constellation of symptoms that include ovulatory dysfunction, hyperandrogenism, and polycystic morphology of the ovaries, as observed through ultrasound imaging [122]. Ovulatory dysfunction manifests as irregular menstrual cycles or anovulation, leading to fertility challenges. Hyperandrogenism results in elevated levels of androgens, causing clinical signs such as hirsutism, acne, and alopecia. Beyond reproductive issues, it is associated with metabolic complications such as insulin resistance, obesity, dyslipidemia, and an increased risk of developing type 2 diabetes and cardiovascular disease. The prevalence of PCOS is exacerbated by a multitude of lifestyle, occupational, and environmental factors [123]. Lifestyle choices such as poor diet, physical inactivity, and obesity contribute significantly to the insulin resistance and hormonal imbalances associated with PCOS.

Dysbiosis in the gut microbiome is linked to increased intestinal permeability, chronic inflammation, and insulin resistance, all of which contribute to the hormonal imbalances seen in PCOS. Targeting gut microbiota with therapies such as probiotics, prebiotics, or fecal microbiota transplantation could provide promising treatment strategies for managing PCOS and its associated metabolic disturbances [124]. Additionally, dysbiosis in PCOS is associated with comorbidities such as type 2 diabetes, non-alcoholic fatty liver disease, and cardiovascular disease [125]. Key microbial metabolites, such as SCFAs, lipopolysaccharides (LPSs), and bile acids, are implicated in these processes, potentially exacerbating PCOS symptoms [126]. Tremellen and Pearce proposed a novel hypothesis regarding the development of PCOS, suggesting that dietary habits play a crucial role in its pathogenesis [127]. According to their model, an unhealthy diet induces dysbiosis of the gut microbiota, leading to increased permeability of the gastrointestinal mucosa. This compromised intestinal barrier allows LPSs from Gram-negative bacteria to translocate into the bloodstream. The presence of LPSs in the systemic circulation activates the immune system and disrupts insulin receptor signaling. Elevated insulin levels, along with increased androgen production, interfere with the normal growth and maturation of ovarian follicles, resulting in impaired follicular development and the characteristic symptoms of PCOS. In this way, alterations in the host’s gut microbiota can activate the immune system and disrupt insulin receptor function. This disruption leads to hyperinsulinemia, which subsequently increases androgen production in the ovaries and hinders the development of normal ovarian follicles [128]. In the context of PCOS, a condition characterized by hyperandrogenism, ovulatory dysfunction, and polycystic ovaries, the gut microbiota plays a significant role in influencing hormonal balance and metabolic processes. Lactic acid bacteria can alleviate PCOS in rat models by increasing the levels of SCFAs through the regulation of sex hormones associated with the gut microbiota [129]. These disturbances create a vicious cycle where PCOS exacerbates gut health and vice versa, highlighting the potential of targeting gut microbiota and metabolites as therapeutic interventions to alleviate PCOS symptoms. Acetate administration in letrozole-induced PCOS rats leads to a reduction in hypothalamic inflammation, the normalization of metabolic and endocrine profiles, and improvements in insulin sensitivity and lipid levels. The study suggests that acetate exerts its beneficial effects through the modulation of NrF2/HIF1-α pathways, ultimately alleviating the lipotoxic and pro-inflammatory damage caused by PCOS [130].

**Figure 3:**
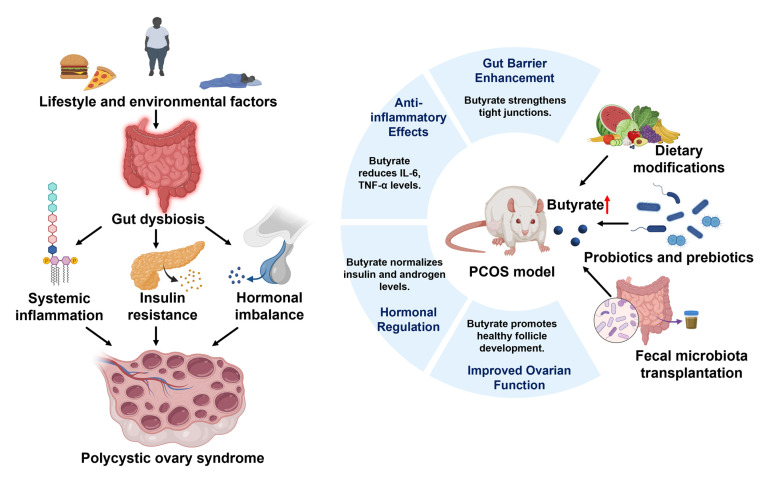
The role of gut dysbiosis and butyrate in the pathogenesis and potential treatment of polycystic ovary syndrome (PCOS). On the left, lifestyle and environmental factors such as poor diet, obesity, and lack of exercise contribute to gut dysbiosis, which leads to systemic inflammation, insulin resistance, and hormonal imbalance. These interconnected factors drive the development of PCOS, characterized by polycystic ovaries and reproductive dysfunction. On the right, therapeutic approaches to enhance butyrate levels—including dietary modifications, probiotics, prebiotics, and fecal microbiota transplantation—are illustrated in a PCOS animal model. Butyrate exerts beneficial effects on PCOS symptoms through various mechanisms. These findings highlight butyrate’s potential as a therapeutic agent for managing PCOS, targeting both metabolic and reproductive abnormalities associated with the syndrome. Upward-pointing arrows indicate the upregulation of butyrate levels as a result of the three interventions shown.

Butyrate plays a particularly vital role in reducing symptoms of PCOS compared to other SCFAs like acetate and propionate [131]. In the context of a butylated starch diet, butyrate significantly improves metabolic and reproductive outcomes in PCOS animal models. Unlike acetate and propionate, butyrate affects peripheral organs indirectly via gut–brain hormonal pathways, offering unique benefits for PCOS treatment. Butyrate has been shown to alleviate PCOS symptoms by acting on the gut–brain axis, particularly through mechanisms involving the regulation of G-protein-coupled receptors such as GPR41. It promotes the secretion of gut hormones, which plays a key role in reducing pathological changes in the ovaries. In the study regarding *Dendrobium officinale* polysaccharide (DOP) and its effects on PCOS, butyrate plays a key role in the gut–brain–ovary axis [132]. The polysaccharide itself is not directly absorbed in the stomach or small intestine; instead, it is degraded by gut microbiota into SCFAs, including butyrate. DOP enhances the diversity of butyrate-producing bacteria, leading to higher levels of butyrate in the gut, which positively impacts endocrine function and ovarian health by interacting with GPR41. In a study investigating the effects of soy isoflavones, resistant starch, and antibiotics on PCOS symptoms in letrozole-treated rats, it was observed that resistant starch plays a key role in elevating butyrate levels in the gut [133]. Butyrate production was significantly enhanced in groups treated with resistant starch, contributing to improved gut barrier function by increasing the expression of gut barrier markers like occludin. Additionally, the combination of soy isoflavones and resistant starch effectively reduced the severity of menstrual irregularity and polycystic ovaries, indicating the potential of dietary interventions in alleviating PCOS-like reproductive symptoms. Furthermore, the increased levels of butyrate following *Lactiplantibacillus plantarum* CCFM1019 administration highlight the potential of butyrate in restoring hormonal balance and improving ovarian function [9]. In a study focused on the effects of butyrate in a PCOS rat model, butyrate was shown to have significant protective effects against renal inflammation and fibrosis. The mechanism involves the suppression of SDF-1 and the subsequent attenuation of inflammation, apoptosis, and fibrotic processes in the kidneys [134]. These findings suggest that butyrate not only improves metabolic disturbances but also restores renal function by mitigating PCOS-related kidney damage. Elevated concentrations of pro-inflammatory cytokines and chemokines, such as interleukin-6 (IL-6) and tumor necrosis factor-alpha (TNF-α), have been found in women with PCOS [135]. Lin et al. discovered that, in a rat model of PCOS, the absorption of SCFAs was reduced [136]. As a result, fecal SCFA concentrations were elevated and showed a positive correlation with increased levels of pro-inflammatory cytokines, specifically IL-6 and TNF-α. These inflammatory mediators play a crucial role in disrupting normal ovarian function by interfering with insulin signaling pathways and promoting insulin resistance. Butyrate has been shown to alleviate inflammation in granulosa cells by regulating mRNA modifications via the METTL3-mediated N6-methyladenosine (m6A) pathway, specifically targeting the FOSL2 gene in the context of PCOS [137]. It reduces the m6A modification of FOSL2, thereby decreasing its expression and leading to a reduction in inflammatory cytokines such as IL-6 and TNF-α. Collectively, these studies highlight butyrate’s multifaceted role in ameliorating PCOS symptoms by modulating gut microbiota composition, enhancing gut barrier function, reducing inflammation, and restoring hormonal balance (Figure 3). This underscores the potential of butyrate as a therapeutic agent in PCOS treatment, paving the way for novel interventions which target metabolic, inflammatory, and reproductive dysfunctions associated with this syndrome.

### 4.5. Endometriosis

Endometriosis is a chronic inflammatory disease and is among the most prevalent gynecological disorders worldwide, affecting approximately 5% of women of reproductive age. Endometriosis is characterized by the presence of endometrial glandular and stromal tissues outside the uterine cavity, commonly on the ovaries, fallopian tubes, and peritoneal surfaces [138]. These ectopic endometrial tissues respond to estrogen-dependent signals similar to the native endometrium, undergoing cyclical growth and proliferation, which can lead to pain and infertility. Recent studies suggest that the malignant potential of endometriosis is closely linked to its pro-inflammatory microenvironment, which promotes neovascularization and contributes to the accumulation of somatic mutations in tumor suppressor genes, such as *p53* and *PTEN*, as well as oncogenes like *BCL-2* [139]. These alterations disrupt normal tissue architecture, creating conditions conducive to malignant transformation. Despite various treatment options, there is currently no definitive and universally effective treatment for endometriosis. The complex pathophysiology of the disease, involving hormonal imbalances, immune system dysregulation, inflammation, and possibly genetic and environmental factors, presents challenges in developing targeted therapies. In addition to presenting gynecological symptoms, up to 90% of individuals diagnosed with endometriosis also report experiencing gastrointestinal disturbances [140].

The recent study found that a reduction in butyrate-producing bacteria was associated with increased inflammation and immune cell activation in endometriotic mice [141]. The results suggest that butyrate plays a key role in modulating immune responses and may help counteract the inflammatory processes in endometriosis, highlighting its potential as a therapeutic target. Restoring gut microbiota balance and increasing SCFA production may serve as promising therapeutic strategies for managing endometriosis, highlighting the gut microbiota’s potential role in both diagnosis and treatment [142]. Butyrate was shown to significantly reduce the size and proliferation of endometriotic lesions in mice, likely through the activation of G-protein-coupled receptors (GPR43 and GPR109A) and the inhibition of HDAC activity [143]. Additionally, butyrate modulated the expression of the tumor suppressor RAP1GAP, leading to the suppression of the RAP1 signaling pathway, which is critical for cell proliferation and lesion growth, highlighting butyrate’s therapeutic potential in managing endometriosis. Collectively, these findings emphasize butyrate’s significant therapeutic potential in endometriosis by modulating immune responses, reducing inflammatory processes, and inhibiting lesion proliferation. This underscores the importance of targeting gut microbiota and SCFA production as innovative strategies for managing endometriosis, warranting further research into butyrate-based interventions to develop more effective and targeted therapies for this complex disease.

## 5. Future Research Directions

While butyrate’s role in gynecological diseases has been studied in terms of its anti-inflammatory, apoptotic, and metabolic effects, the precise molecular mechanisms underlying its therapeutic potential remain incompletely understood. The interaction between butyrate and hormonal signaling pathways, such as estrogen and progesterone receptor pathways, remains largely unexplored [144]. Since hormonal imbalance is a hallmark of conditions like PCOS and endometriosis, investigating how butyrate modulates these signaling pathways could uncover novel therapeutic targets. Moreover, the specific role of butyrate in bridging immune system dynamics and reproductive health remains unclear. Future studies should investigate how butyrate influences immune cell populations, such as macrophages and T cells, within the female reproductive tract and whether this cross-talk impacts disease progression or resolution [145]. Furthermore, emerging evidence suggests that butyrate can influence the metabolic reprogramming of cancer cells [146]. However, its specific role in altering the tumor microenvironment in gynecological cancers, such as the switch between glycolysis and oxidative phosphorylation, remains an open question. Future research could focus on identifying metabolic vulnerabilities in tumors that could be targeted with butyrate-based therapies.

Despite its therapeutic potential, butyrate’s clinical application is limited by its rapid absorption and metabolism, which result in low systemic availability and reduced efficacy in targeting specific tissues. Current research on butyrate delivery systems, while promising, remains in its infancy and requires further exploration. Advances in encapsulation have shown potential for protecting butyrate from premature metabolism and enhancing its stability [147,148]. However, the effectiveness of these systems in gynecological disease models remains largely unexplored. Innovations in tissue-specific targeting, such as the use of ligand-conjugated nanoparticles or prodrug formulations activated in gynecological tissues, could significantly enhance butyrate’s therapeutic potential. Developing delivery systems that exploit the unique microenvironment of the female reproductive system, such as pH sensitivity or enzyme-specific activation, could improve localization and reduce off-target effects.

The integration of butyrate with existing chemotherapeutic and hormonal therapies offers significant potential to enhance treatment efficacy in gynecological diseases. However, its mechanisms of synergy and its role in overcoming limitations such as chemoresistance remain underexplored. Chemoresistance poses a major challenge in the treatment of gynecological cancers, including ovarian and cervical cancer. Butyrate’s ability to modulate epigenetic regulators and restore apoptotic pathways may play a critical role in sensitizing cancer cells to chemotherapeutic agents like cisplatin or paclitaxel [149]. Moreover, hormonal imbalances are central to conditions like PCOS and endometrial hyperplasia. Butyrate’s potential to modulate estrogen and androgen receptor activity, as well as its influence on hormonal signaling pathways, could enhance the efficacy of hormonal therapies [150,151]. Further studies are needed to determine the compatibility and timing of butyrate administration alongside hormonal agents. Finally, large-scale trials should be conducted to assess the safety, efficacy, and optimal dosing of combination therapies involving butyrate. Trials could explore the impact of butyrate in enhancing the therapeutic index, reducing side effects, and improving patient outcomes when used in combination with standard treatments for gynecological diseases.

## 6. Conclusions

Probiotics and SCFAs have emerged as promising therapeutic agents due to their ability to enhance gut health, reduce inflammation, and support microbial balance. Butyrate, known for its protective role in intestinal health, also exhibits anticancer properties through mechanisms like HDAC inhibition, which promote cell differentiation and apoptosis. However, its therapeutic application is hindered by rapid metabolism, prompting the exploration of alternatives such as tributyrin and dietary interventions [152]. Dietary patterns rich in fiber, such as the Mediterranean diet, have demonstrated potential in naturally boosting butyrate production, thereby alleviating the inflammation and hormonal imbalances associated with conditions like endometriosis, PCOS, and gynecological cancers [153]. While these strategies offer promise, the precise mechanisms by which SCFAs influence gynecological health remain underexplored. Further research is essential to elucidate the complex interactions between SCFAs, gut microbiota, and the female reproductive system. Such insights could pave the way for novel microbiota-based therapeutic approaches to improve reproductive health and overall quality of life for women affected by these conditions.

## Data Availability

No new data were created or analyzed in this study. Data sharing is not applicable to this article.
